# Bioaccessibility of Shore Magic^®^ collagen, a low-molecular-weight collagen supplement, in different *in vitro* barrier models

**DOI:** 10.1016/j.heliyon.2018.e00821

**Published:** 2018-09-26

**Authors:** Marcel Benadiba, Raphael Serruya, Yehoshua Maor

**Affiliations:** Phytor Lab for Drug Development, Hadassah Medical Center Hebrew University Biotechnology Park (JBP), Ein Kerem Campus, Jerusalem 91120, Israel

**Keywords:** Biochemistry, Biotechnology, Cell biology

## Abstract

Hydrolyzed collagen consists of peptides, which exert important biological functions in different body systems. This study aimed at testing the biological effects of a low molecular weight collagen (LMWC), namely Shore Magic® Collagen (SMC), in a series of *in vitro* assays and three different *in vitro* barrier models with translational significance. We also compared SMC's biological activities with its trypsinized form (TSMC). SMC enhanced migration in both epithelial and endothelial cells; and increased the adhesion of epithelial cells, but surprisingly not of endothelial cells. It also diminished the tightness in the gut and blood-brain barriers *in vitro* while TSMC did not. SMC induced both neurogenesis and BJ epithelial cell proliferation of cells growing below the *in vitro* barriers. In conclusion, the intact form of SMC shows enhanced bioavailability and efficiency compared with TSMC.

## 1 Introduction

Proteins comprise almost 15% of the human body. Collagen is the major protein of the extracellular matrix (ECM) and is the most abundant in mammals, representing 70%–80% of skin proteins (Brett, 2008). Collagen acts as a structural scaffold in tissues. The central feature of all collagen molecules is their rigid, triple-stranded helical structure (Brett, 2008). Types I, II, and III are the main types of collagen found in connective tissue and constitute 90% of all collagen in the body (Brett, 2008).

Collagen has long been used as a nutritional supplement. However, its digestion, degradation in the gastrointestinal tract, and bioavailability are concerns that raise questions about its effectiveness and uses in the clinic. The development of more collagen supplements brought to the market hydrolyzed collagen with higher bioavailability. Hydrolysate Collagen (HC) consists of small peptides with a low molecular weight (lower than 5.000 Da.), which are produced from gelatinization and enzymatic hydrolysis of collagen derived from animal tissues (Bello and Oesser, 2006). Collagen-derived peptides control many cellular functions, including cell shape and differentiation migration, and synthesis of many proteins (Hynes, 1992). Bioactive peptides from hydrolyzed collagen have been shown to play a significant therapeutic role in human health and disease. Of note, a study using a nutritional supplement consisting of hydrolyzed collagen, hyaluronic acid, vitamins, and minerals demonstrated a reduction in skin dryness, wrinkles, and nasolabial fold depth, along with a significant increase in collagen density and skin firmness (Sibilla and Borumand, 2014).

In the present study, we aimed at evaluating the influence of low molecular weight peptides derived from hydrolyzed collagen from a nutritional supplement, branded Shore Magic® Collagen (SMC), on cellular migration, adhesion, and proliferation. Secondly, we aimed at evaluating the ability of this collagen supplement to cross membranes in translational significant in *in vitro* models of the gut-brain axis (GBA), gut-epithelial axis (GEA), and blood-brain barrier (BBB), and also to assess the effects on epithelial cells and neurons growing below gut and brain barriers *in vitro*. To compare the reported clinical benefits of the supplement with amino acids (AA) derived from the peptides, we mimicked the degradation of SMC using trypsin, which generates smaller peptides/AA.

## 2 Materials and methods

### 2.1 Reagents and media

All reagents, media and supplements were purchased from Biological Industries – Israel, except where clearly stated in the methods described below.

### 2.2 Cell culture

Adherent human endothelial (human vascular endothelial cells – HUVEC), epithelial (Human Foreskin Fibroblast – BJ or HFF), colorectal (Caco-2), and neuronal (SH-SY5Y) cells were cultured according to standard mammalian tissue culture protocols and sterile conditions. Caco-2 cells were cultured in high glucose Dulbecco's Modified Eagle Medium; SH-SY5Y cells were cultured in a Roswell Park Memorial Institute (RPMI) medium; HUVEC cells were cultured in Endothelial Cell Growth Medium; and BJ and HFF cells were cultured in BIOAMF™-2 Amniotic Fluid & Chorionic Villus Cell Culture Medium. All media—except for the BIOAMF™-2, which is complete—was supplemented with 10% fetal bovine serum, streptomycin (100 μg/ml), penicillin (100 U/ml), and nystatin (12.5 U/ml). Cells were incubated in a 5% CO_2_ containing atmosphere at 37 °C. All tissue cultures were maintained in a 25 cm^2^ NuncTM cell culture treated with EasYFlaskTM (from Thermo Fisher Scientific), and all the media and supplements were obtained from Biological Industries – Israel.

### 2.3 Preparation of trypsinized SMC (TSMC)

Degradation of SMC was tested using an acidic solution (pH 1.8) and the trypsin/EDTA Solution B (0.25% trypsin and 0.05% EDTA in Puck's Saline A) from Biological Industries – Israel. We dissolved 1 mg SMC in 1 ml double distilled water (SDDW) containing 2 μl hydrochloric acid (final pH = 1.8) or in 1 ml of the trypsin-containing solution described above – modified from (Thiede et al., 2000). HPLC analysis (described below) was conducted for evaluating the obtained results after degradation.

### 2.4 Agarose spot assay

One tenth of a gram of SMC + 0.1 g of low-melting-point agarose (Invitrogen) were placed into a 100 ml beaker and diluted into 20 ml phosphate buffer saline (PBS) solution. This solution was heated until boiling, swirled to facilitate complete dissolution, and then allowed to cool. When the temperature reached 40 °C, 90 μl of the agarose-containing SMC solution and the respective control (without SMC) was pipetted into a 1.5 ml Eppendorf tube. Using cut-pipette tips, 10 μl spots of these solutions were placed inside the wells of a six-well plate as rapidly as possible and allowed to cool for ≈5 min at 4 °C. Four spots per well were pipetted; two containing SMC and two containing only PBS. At this point, Aspc1 human pancreatic adenocarcinoma cells were plated into spot-containing wells in the presence of a 10% FBS cell culture medium and allowed to adhere for four hours. Cells were then transferred into the cell culture medium with 0.1% FBS, returned to the incubator overnight, and analyzed by microscopy the next morning. Imaging was performed on a Primovert™ inverted microscope equipped with a Zeiss® Axiocam 105 Color with a 10X or 20X objective, and for each spot we recorded the field that contained the highest apparent number of motile cells penetrating furthest underneath the agarose spot.

### 2.5 Wound healing assay

Twenty-four well plates were incubated with 1, 3, and 5 mg/ml SMC containing solutions. One hour later, HUVEC, BJ, or HFF cells were seeded at a concentration of 5 × 10^4^/well (n = 4) and allowed to grow in 10% FBS containing the respective media until they reached the confluence of 90%. For migration studies, a 1 mm-deep scratch was made across the cell layer using a sterile pipette tip. The old media containing cellular debris was replaced with fresh media. Plates were photographed immediately after scratching and again after 24 hours, at the identical location of the initial image. The Primovert™ inverted microscope equipped with a Zeiss® Axiocam 105 Color with a magnification factor of 10 was used to collect the pictures. The quantification analysis was performed using ImageJ^®^ software, where the area occupied by the cells was measured as a percentage and plotted using Microsoft Excel. For the cell proliferation assay, the Trypan blue exclusion method was used for counting viable cells after they reached the desired confluence. Cell viability was calculated as the number of viable cells divided by the total number of cells within the grids on the hemocytometer and plotted using Excel.

### 2.6 Adhesion assay

As the wound healing assay, SMC 1, 3 and 5 mg/ml in serum-free medium was pre-incubated in a 24-well plate. One hour later, the human epithelial kidney (HEK) cell line was seeded at a density of 5 × 10^4^/well (n = 4). The next day, a trypsin/EDTA solution B containing 0.25% trypsin and 0.05% EDTA in Puck's Saline A (Biological Industries) was used to detach the cells from the plate. After removing the medium, we added the trypsin solution to the cells and the stopwatch was triggered. When the cells were entirely detached the stopwatch was stopped and the time recorded.

### 2.7 MTT assay

Briefly, cells were seeded in 96-well plates at a density of 1 × 10^4^ cells/well (n = 4). After overnight stabilization, cells were exposed to varying concentrations (1–500 μg/ml) of SMC or TSMC. Then, plates were incubated in a humidified atmosphere containing 5% CO_2_ in air at 37 °C. After 24 hours, the media were removed, and all cells were incubated with serum-free media containing 0.5 mg/ml 3- [4, 5-dimethyl-thiazol-2-yl] 2, 5 diphenyltetrazolium bromide (MTT; Abcam, ab146345) for two to four hours in the incubator. The MTT purple crystals formed by the viable cells were diluted using isopropanol containing 0.04 mol/L HCl. The quantification was determined by measuring the optical density at 570 nm in an enzyme-linked immunosorbent assay (Spark, Tecan) reader. Data were presented as proportional viability (%) by comparing the treated group with the untreated cells; the viability of which is assumed to be 100%.

### 2.8 Transepithelial electrical resistance (TEER) measurements

Colon cancer (Caco-2) for the GBA or the GEA model, or human umbilical vein endothelial cells (HUVEC) for the BBB model, were plated in a 6.5 mm Transwell with a 0.4 μm pore polycarbonate membrane insert (Millipore®). Epithelial (BJ) or neuroblastoma (SH-SY5Y) cells were plated below the inserts on the bottom of the well in a 24-well polystyrene plate. For more realistic development of the BBB *in vitro*, U87MG astrocyte-derived cells were plated under the surface of the insert. Seventy-two hours after seeding, TEER (transepithelial electrical resistance) was used to determine the monolayer integrity. Twenty-four hours later, the plates were equilibrated at room temperature for one hour. The electrical resistance across the monolayer was measured using an ohmmeter (Millicell®) equipped with probes, positioning one probe inside the filter well and the second in the medium in the growth well. The electrical resistance for each well was recorded. The electrical resistance of a blank insert (without cells) was determined to subtract the background value.

### 2.9 HPLC analysis

HPLC analysis was performed to check the chromatogram profile of SMC and TSMC prior to being used in the biological assays. The samples were filtered in a syringe-driven filter unit (phobic PTFE 0.20 μm, Millipore), and 30 μl was injected using a chromatogram method, which was developed to facilitate HPLC fractionation of a partially purified sample. A Dionex Ultimate 3000 system and a Phenomenex C-18 (4.6 × 250) Luna Column was used. The samples were injected at a flow of 1 ml/min. The final conditions used for analytic HPLC were: at zero min. – 50% methanol, 50% water; at 45 min. – 95% methanol, 5% water; and at 60 min. – 95% methanol, 5% water. The temperature of the column was 37 °C, and UV detection was carried out at 315 nm.

### 2.10 Statistical analysis

We used Student's t-test for statistical analysis in Microsoft Excel. Data were considered significantly different from control when p < 0.05.

## 3 Results and discussion

### 3.1 Effect of SMC on cell adhesion and migration

**Fig. 1:**
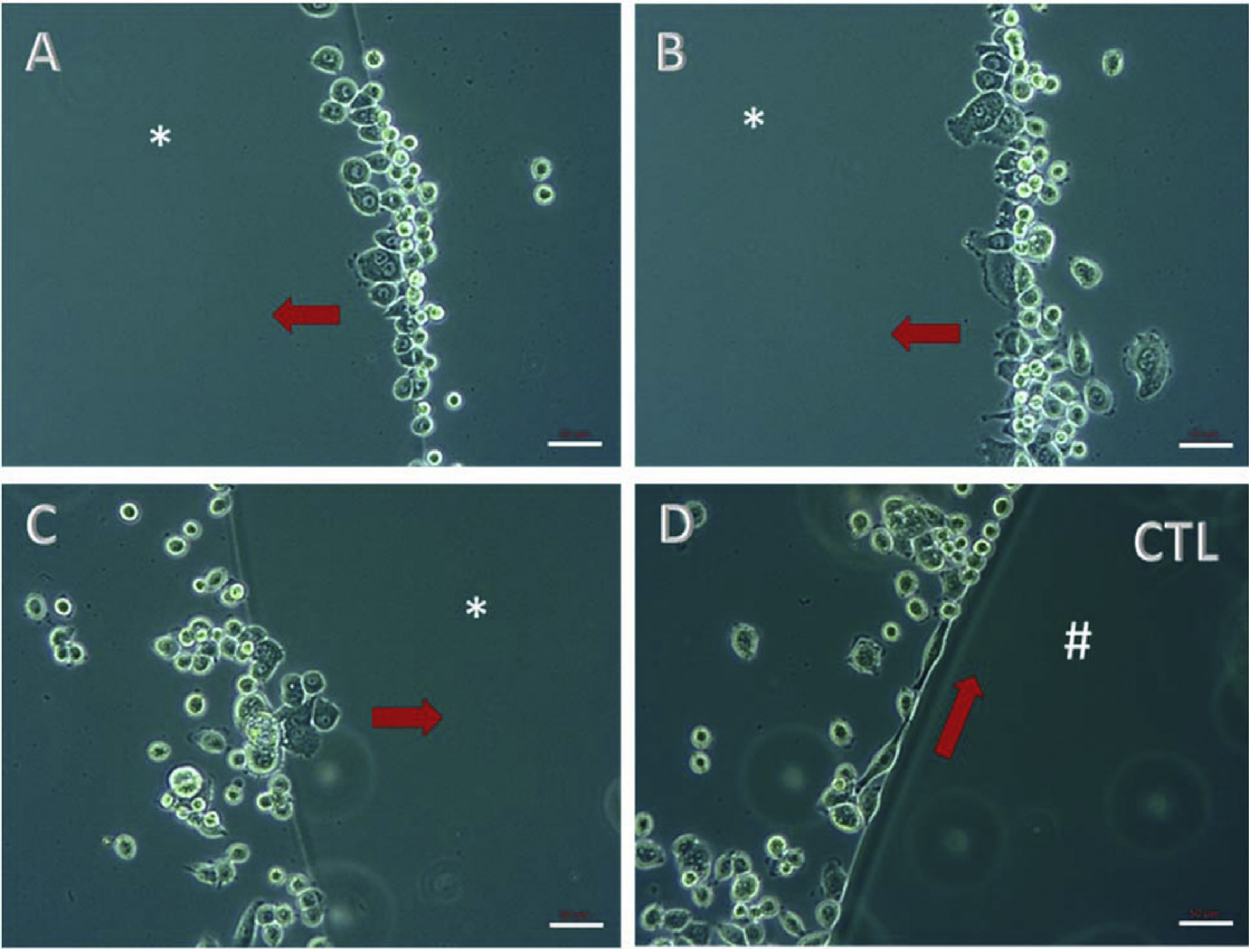
Agarose spot assay. Aspc1 cells were used and the incubation time after plating was 24 h. *drop of agarose containing 0.5% SMC; #drop of agarose without SMC. Different regions are demonstrated as A, B and C micrographs showing the chemotactic effect of SMC. Arrows indicate the direction of migration/invasion. A representative micrograph of control cells (CTL), which did not invade the agarose spot, is showed (D). Scale bar – 50 μm.

Cellular adhesion is a fundamental and well-controlled process that involves the interaction of surrounding extracellular matrix protein components (e.g., collagen) with structural receptors, called integrins (Rudolph and Cheresh, 1990). Integrins allow cells to maintain contact with one another and with structures in the extracellular matrix. They also transmit signals to and from cells. In this way, these molecules play an essential role in sensing the environment and controlling cell shape and motility (Eckes et al., 2010). To our knowledge, metastatic cancer cells have developed the ability to migrate and invade surrounding tissues using all the machinery mentioned above, and, moreover, using collagen as a trail for their motility. Aspc1, pancreatic ductal adenocarcinoma cells, are metastatic cells and were used in the present study as a model *in vitro* to test whether SMC can induce cell migration/invasion, just as collagen does *in vivo*. The results confirmed that although it is a hydrolyzed collagen, SMC can stimulate cells to migrate/invade (Fig. 1). This result may be of concern for cancer patients or for those who are at risk of developing cancer.

**Fig. 2:**
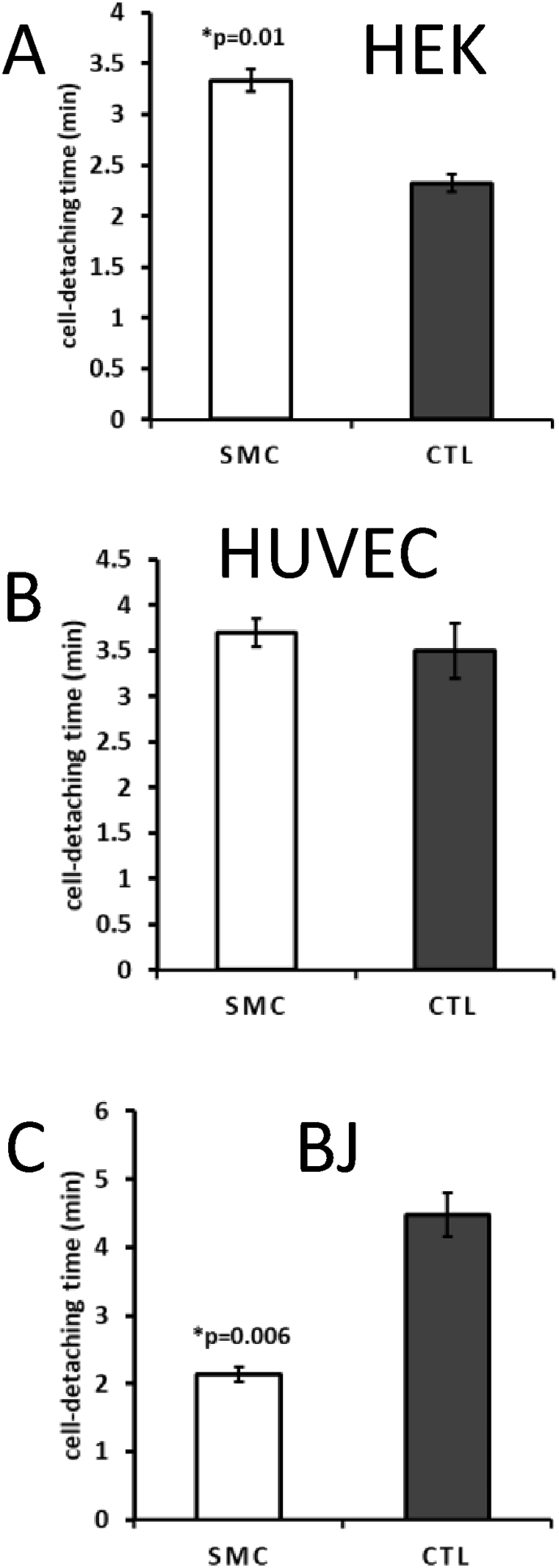
Adhesion assay. The concentration of 1 mg/ml SMC is demonstrated here. A) SMC significantly increased HEK cell adhesion compared to control (CTL). B) No difference was observed on HUVEC cells. C) A very significant decrease in BJ cell adhesion was observed after treatment. The data are mean ± SE (n = 4). Difference between the control and treatment is significant (Student's t-test, p < 0.05). Scale bar – 50 μm.

To validate the adhesion study, HEK293 cell lines (HEK) were used, which are known for their poor attachment capability to plate surfaces. We have tested 1, 3 and 5 mg/ml concentrations. However, since no significant difference was observed on the effect carried out by the higher concentrations tested, we only have reported the results obtained from 1 mg/ml SMC. In the assay, SMC was able to significantly and specifically increase HEK cell attachment compared with control (CTL) (Fig. 2A) and compared with HUVEC cells (Fig. 2B), which possess a relatively high attachment capability. Because of SMC's ability to stimulate epithelial cell migration, control cells (BJ) that were not incubated with SMC were expected to be more attached to the plate and therefore less prone to migrate. This behavior was indeed observed in the experiment using BJ cells (Fig. 2C).

**Fig. 3:**
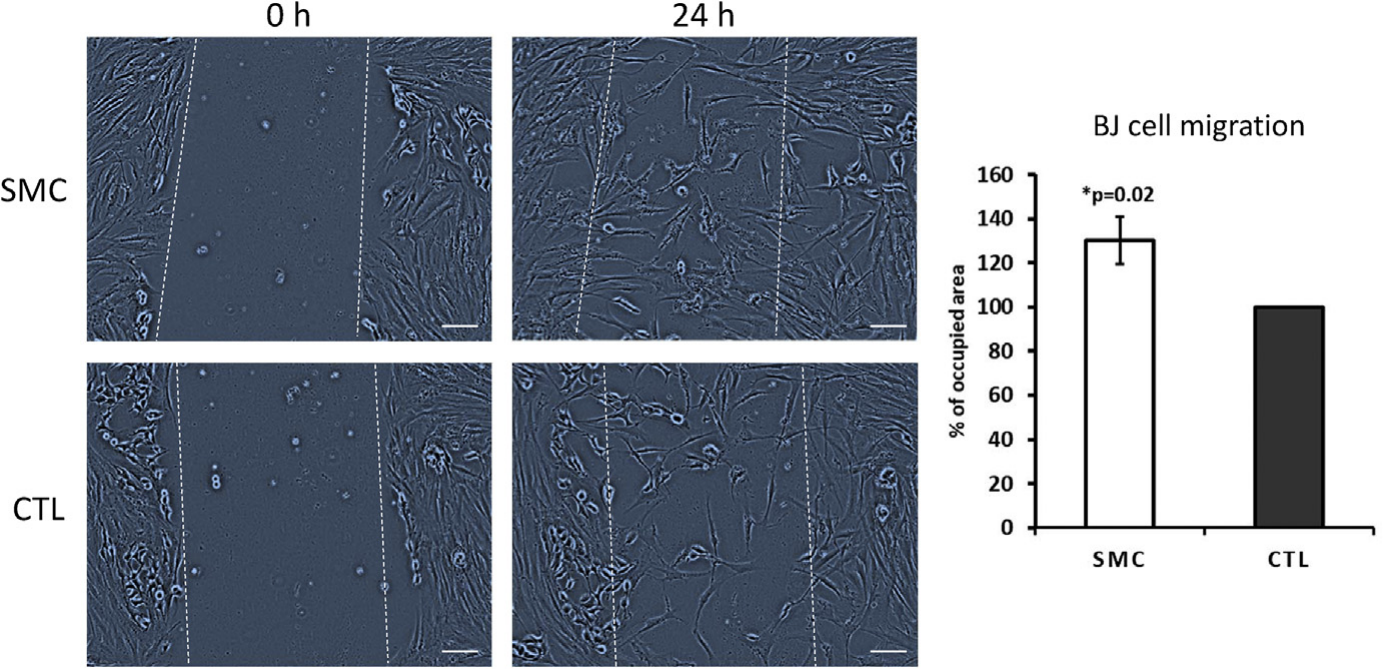
Wound healing assay on BJ cells. A pre-incubation using 1 mg/ml SMC was performed. The data are mean ± SE (n = 4). Difference between the control and treatment is significant (Student's t-test, p < 0.05).

**Fig. 4:**
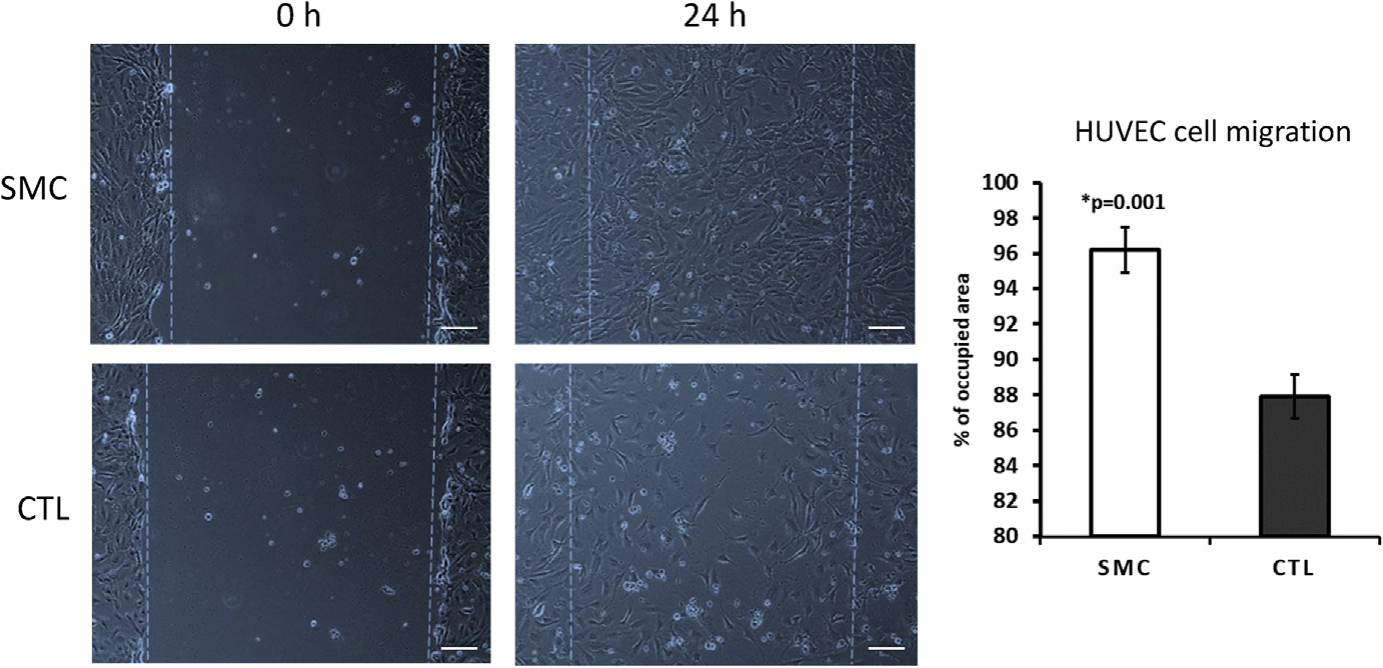
Wound healing assay on HUVEC cells. A pre-incubation using 1 mg/ml SMC was performed. The data are mean ± SE (n = 4). Difference between the control and treatment is significant (Student's t-test, p < 0.05).

Concerning the migratory ability of BJ and HUVEC cells, SMC was able to increase their performance significantly in comparison with control (Figs. 3 and 4). The experiments were performed by pre-incubating concentrations of 1, 3 and 5 mg/ml SMC for one hour prior to plating. Here we show only the significant effective SMC concentrations. Namely, 3 mg/ml for BJ cells and 1 mg/ml for HUVEC cells. Afterwards, the serum-free medium was removed, and a fresh FBS containing medium with the respective cells was added and incubated for an additional 24 hours. During the previous one hour pre-incubation period, SMC is expected to attach to the polypropylene surface of the well, functioning as a trail for the cells. As demonstrated in Figs. 3 and 4, the cells were able to migrate through the wound. Typically, in an *in vivo* microenvironment, five to seven days after the initial injury, fibroblasts migrate to the wound site and secrete new collagen. Keratinocytes (epithelial cells that produce keratin) *per se* migrate from the wound edge and form a thin epithelial cell layer to close the wound (Chen et al., 2014; Singer and Clark, 1999). In this assay, we did not use a co-culture system with fibroblast. Thus, our findings demonstrated that SMC is indeed sufficient for BJ and HUVEC (epithelial and endothelial cells, respectively) to accelerate the process of wound healing.

When cells are migrating, they are not proliferating. Therefore, cells should be more readily released from contact sites. Interestingly, in an assay performed in a 24-well plate, it was visually observed that the center of the wells in the control samples was less confluent than that treated with SMC (data not shown). In the wake of that observation, we used the Trypan blue exclusion method to check the number of live cells in each sample. Surprisingly, we observed that even with fewer cells in the center of the well, there were significantly more cells in the control samples compared with the treated ones. This result strongly suggests that the cells from the treated samples were more biochemically stimulated to migrate and spread in the well, especially in the center, rather than to proliferate. Also, the treatment usually flows more to the middle of the well, which also helps to explain the observed behavior. Conversely, the control samples had clusters of proliferating cells in the borders of the well, which is usually the initial site where the cells naturally flow after the seeding process.

### 3.2 In vitro analysis of the integral SMC versus trypsinized SMC (TSMC) on cell proliferation

Daily intake of collagen hydrolysate has been reported to improve skin and joint conditions, and these beneficial effects are exerted by hydroxyproline (Hyp) containing peptides, which were detected in human blood soon after ingestion (Shigemura et al., 2017). Daily intake of collagen hydrolysate for an extended period can change compositional rate of Hyp-containing peptides in human blood. Importantly, Shigemura et al. suggested in their study that long-term ingestion of collagen hydrolysate might change exo- or endo-type protease activity in the digestive tract, with beneficial effects not only on the GI tract but also in axes such as GBA and GEA. The models utilized in the current study provide a plausible *in vitro* explanation of the beneficial effects of collagen hydrolysate on different body systems.

**Fig. 5:**
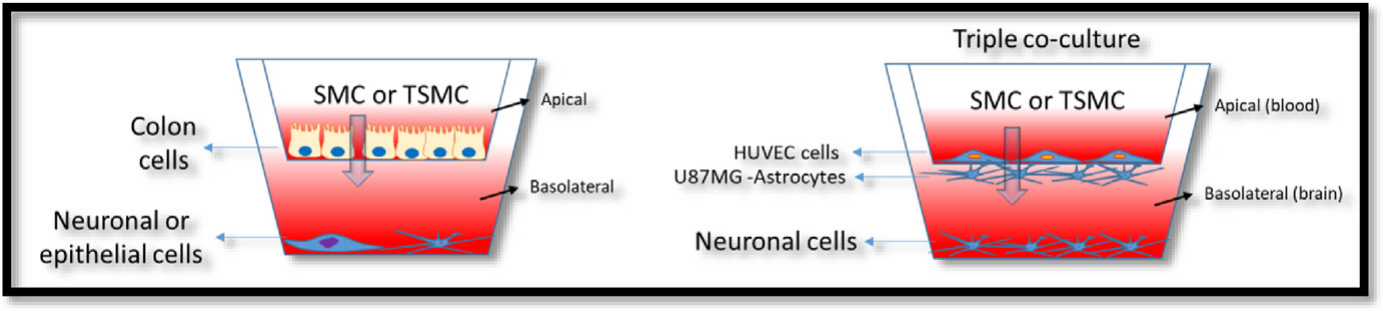
Transwell model illustration. A scheme of dual-chamber or transwell models of the gut-brain axis and gut-epithelial axis (left), and the triple co-culture model of the blood-brain barrier (BBB) (right) is shown. The apical and the basolateral side are indicated in the figure. The effects of trypsinized SMC (TSMC) versus SMC were compared.

In the following assays, we investigated the ability of SMC not attached to any surface, as opposed to the previous experiments. The goal was to compare SMC and its effect on different body tissues such as skin and brain, to determine the degree of effectiveness in generating a biological response for both biological targets in an axis model (Fig. 5). We sought to determine whether the biomedical benefits of orally-taken SMC derive from SMC itself or if the intake of amino acids that compose collagen could also lead to the beneficial effects observed by the ingestion of collagen supplements.

**Fig. 6:**
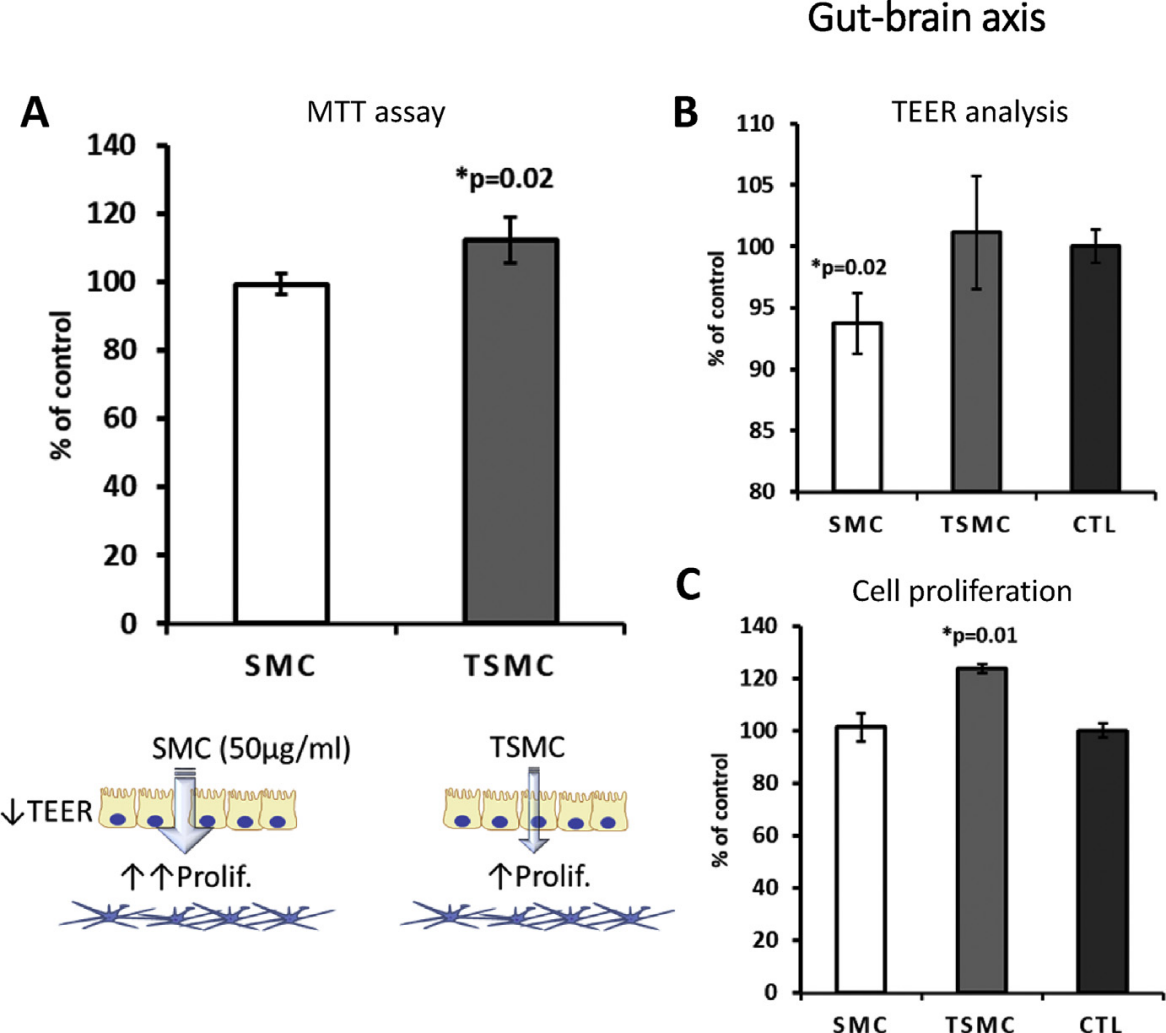
Gut-brain axis model. 15 μg/ml was the concentration used for both SMC and TSMC. A) MTT assay showing significant increase in neuronal cell viability after TSMC treatment. B) A significant decrease on TEER after SMC treatment was observed in comparison with control and TSMC. C) On the basolateral side an increase in neuronal cell proliferation was observed after TSMC treatment.

**Fig. 7:**
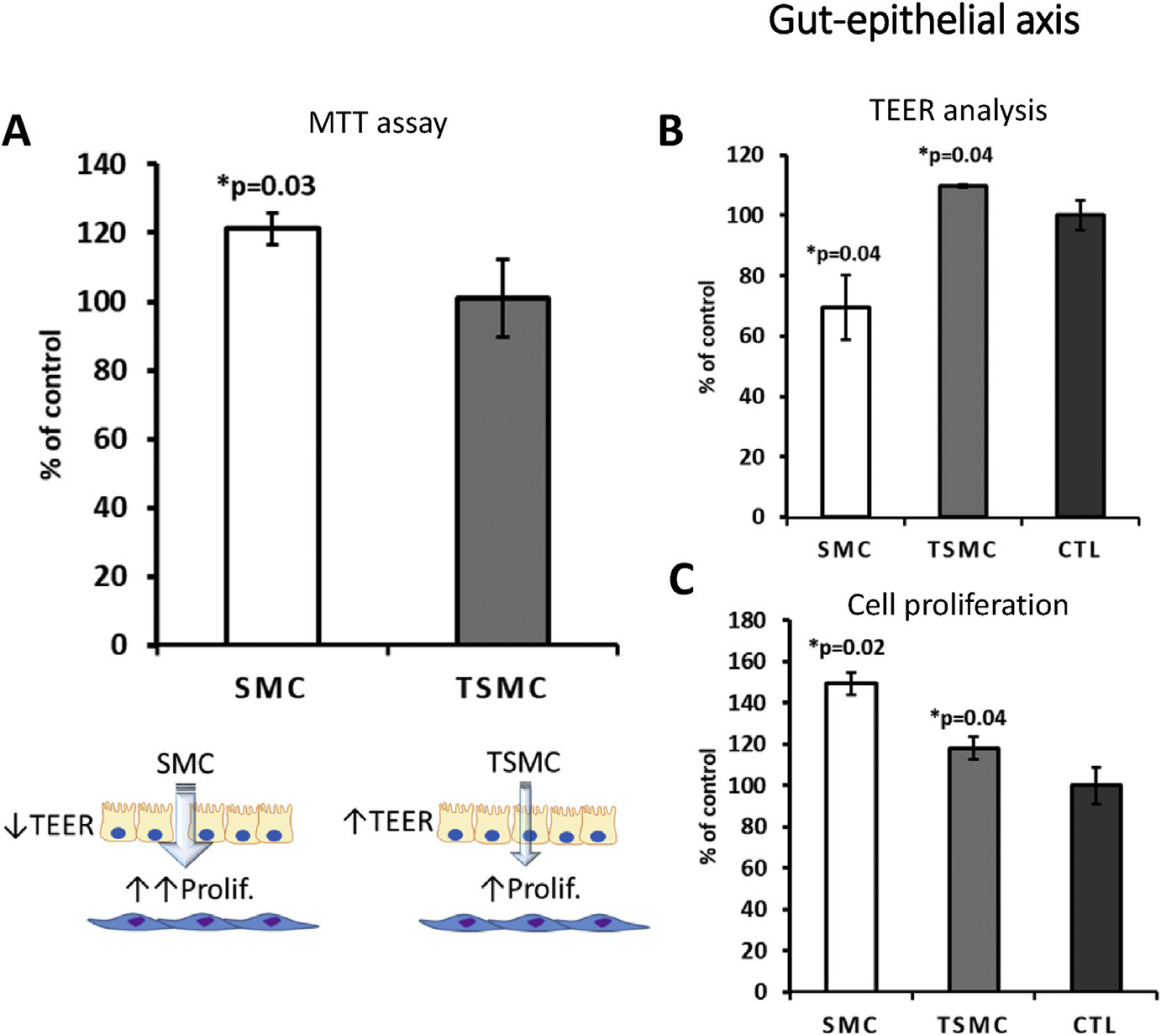
Gut-epithelial axis model. 15 μg/ml was the concentration used for both SMC and TSMC. A) MTT assay showing a significant increase in BJ cell viability after SMC treatment. B) SMC caused a significant decrease on TEER, whereas an increase was observed after TSMC treatment. C) BJ cell proliferation was increased by both SMC and TSMC treatment in comparison with control. Note that SMC more efficiently induced BJ cell proliferation in comparison with control.

**Fig. 8:**
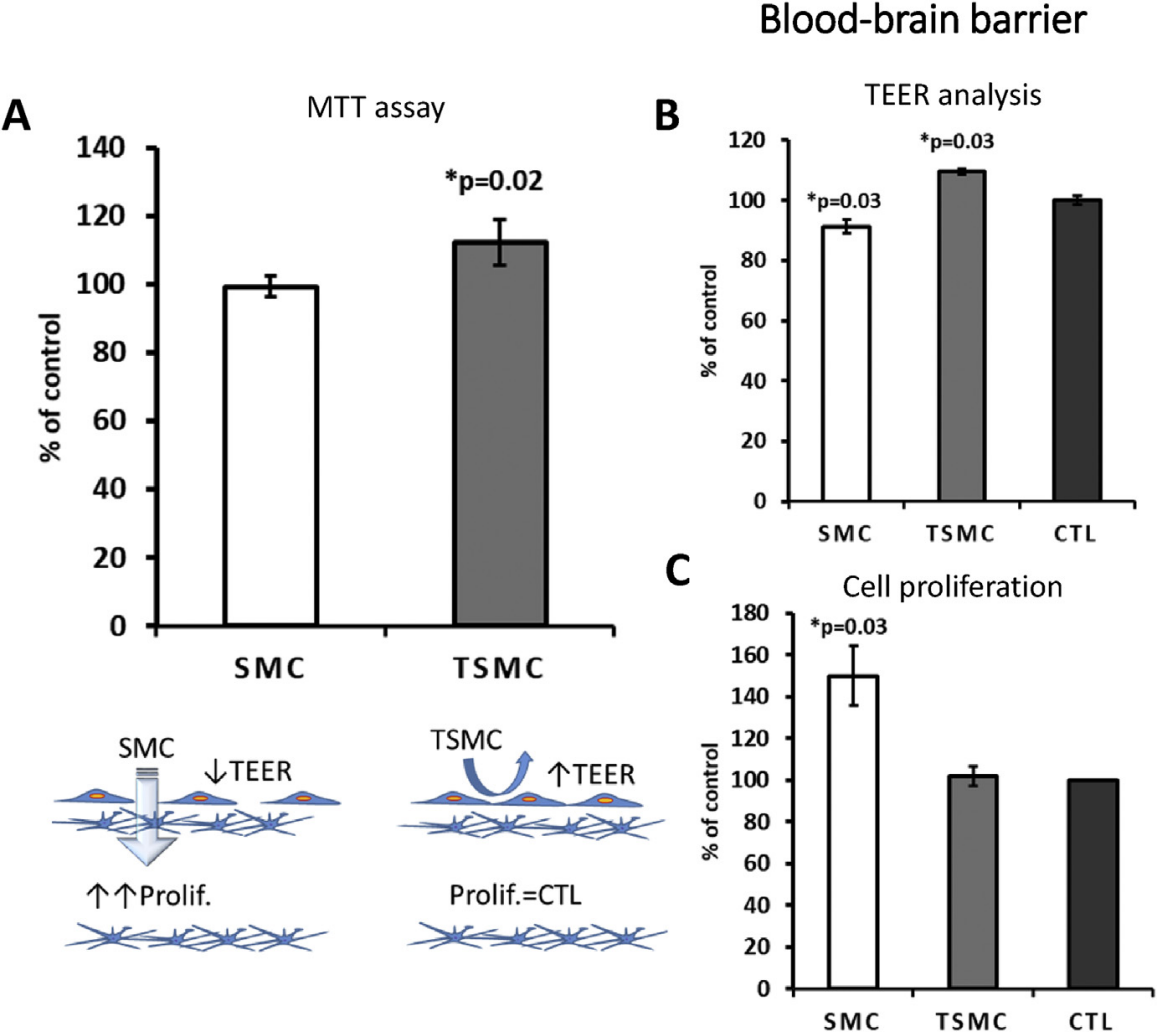
Blood-brain barrier model. 15 μg/ml was the concentration used for both SMC and TSMC. A) For comparison purposes, the same MTT assay showed in Fig. 6 is showed again. B) SMC caused a significant decrease on TEER, whereas an increase was observed after TSMC treatment. C) On the basolateral side a significant increase in neuronal cell proliferation after SMC but not after TSMC treatment was observed. The data are mean ± SE (n = 4). Difference between the control and treatment is significant (Student's t-test, p < 0.05).

Using MTT (3-(4,5-dimethylthiazol-2-yl)-2,5-diphenyltetrazolium bromide), tetrazolium reduction assay, we observed that TSMC—but not SMC—was able to stimulate neuronal cell monolayer viability, measured in a 96-well plate (Fig. 6A). Moreover, using a GBA model *in vitro* (Foster and McVey Neufeld, 2013; Kang and Kim, 2016), it was observed that TSMC increased the neuronal cell number in the basolateral side, suggesting that TSMC is indeed able to cross the gut barrier (Caco-2 cell line) (Fig. 6C). Importantly, SMC, but not TSMC, caused a decrease in the TEER in all barrier models (Figs. 6B, 7B and 8B), suggesting that the tightness of the gut and endothelial cells growing on the inserts was weakened by the treatment. After evaluating a concentration-response curve, the concentration of 15 μg/ml was chosen for all the experiments below, because it did not affect the viability of the cells studied. A higher concentration of 30 μg/ml SMC caused a significant decrease in neuronal cell viability, as observed by MTT analysis (data not shown). BJ cells were used in the GEA model (see below).

**Fig. 9:**
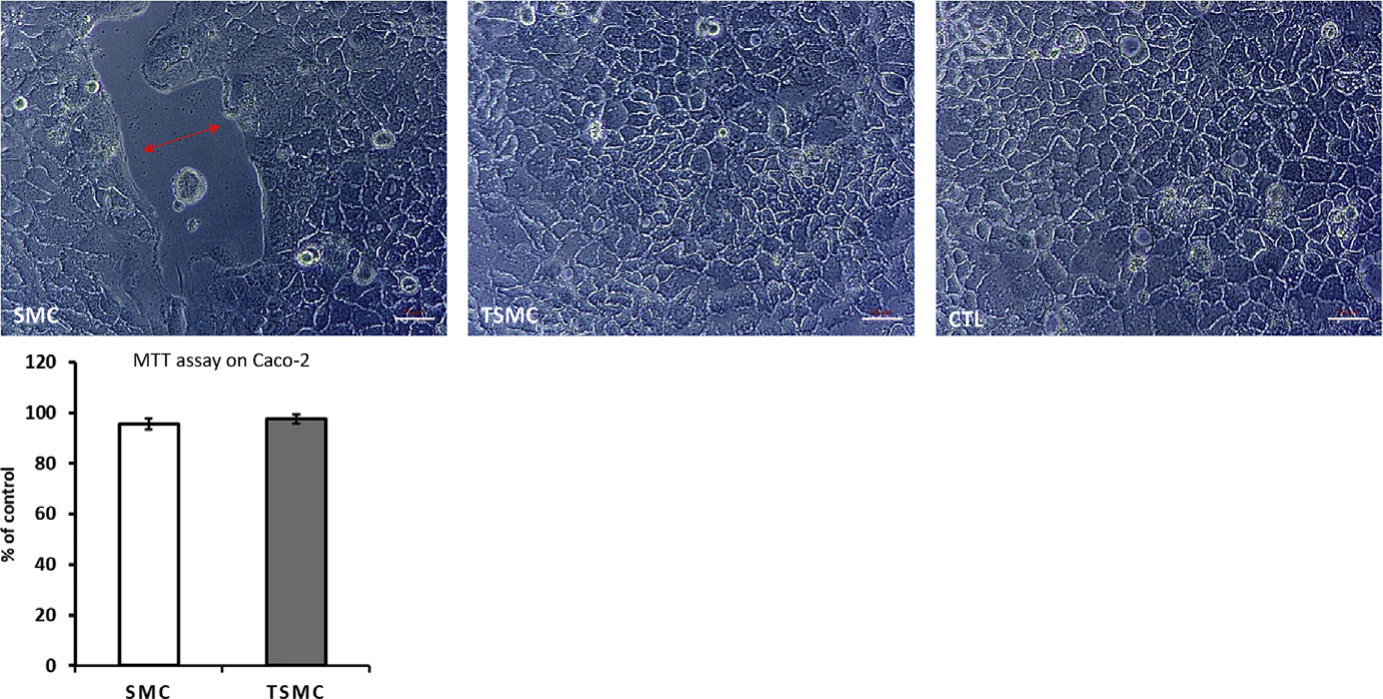
Representative micrographs of Caco-2 cell monolayer. Note the loss of tightness between cells (double-headed arrow) after treatment with 15 μg/ml SMC but not after TSMC or control (CTL). 20X zoom magnification. The graphic below shows that the viability of Caco-2 is not affected. Thus, it is possible to conclude that 15 μg/ml SMC is able to loosen Caco-2 tight junctions without affecting cell viability. The data are mean ± SE (n = 4). Difference between the control and treatment is significant (Student's t-test, p < 0.05). Scale bar – 50 μm.

SMC at a concentration of 8 μg/ml significantly increased BJ cell metabolism measured in a 96-well plate (Fig. 7A). Furthermore, in the GEA model, BJ cells responded significantly to both SMC and TSMC, with an increase in cell proliferation; however, with SMC being more effective (Fig. 7C). As mentioned above, SMC was able to decrease TEER (Figs. 6B and 7B), indicating that loosening the tight junctions of the gut-barrier cells partially explains its proliferative activity on cells in the lower chamber (basolateral side). On the other hand, TSMC caused an increase in TEER (Fig. 7B), which may explain its diminished efficiency in increasing BJ cell proliferation. Noteworthily, no increase or decrease in Caco-2 cell viability (see Fig. 9) after TSMC treatment was observed. This result confirms that Caco-2 may only transport the AA/peptides from the apical to the basolateral side (Goulart et al., 2014) without being affected by the additional AA/peptides.

**Fig. 10:**
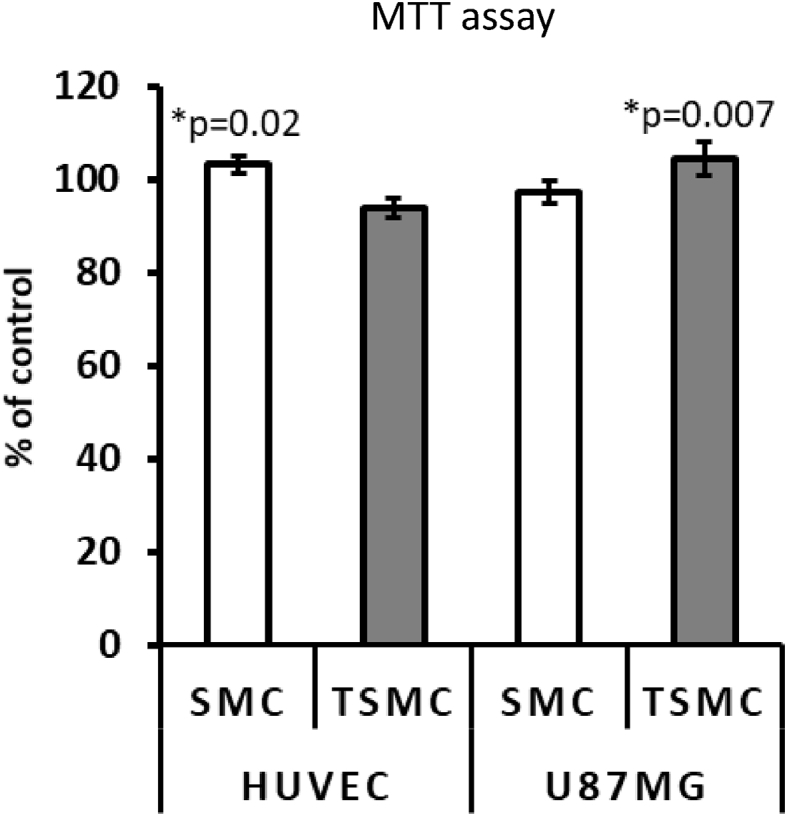
HUVEC and U87MG cell viability. The results showed that the treatment does not damage the cells. Both cell lines are used in the BBB model. Importantly, U87MG cells do not form a monolayer, as they are stellar cells. They are used only to improve the tightness of HUVEC cells on the BBB model, akin to the physiological situation *in vivo*. The data are mean ± SE (n = 4). Difference between the control and treatment is significant (Student's t-test, p < 0.05).

**Fig. 11:**
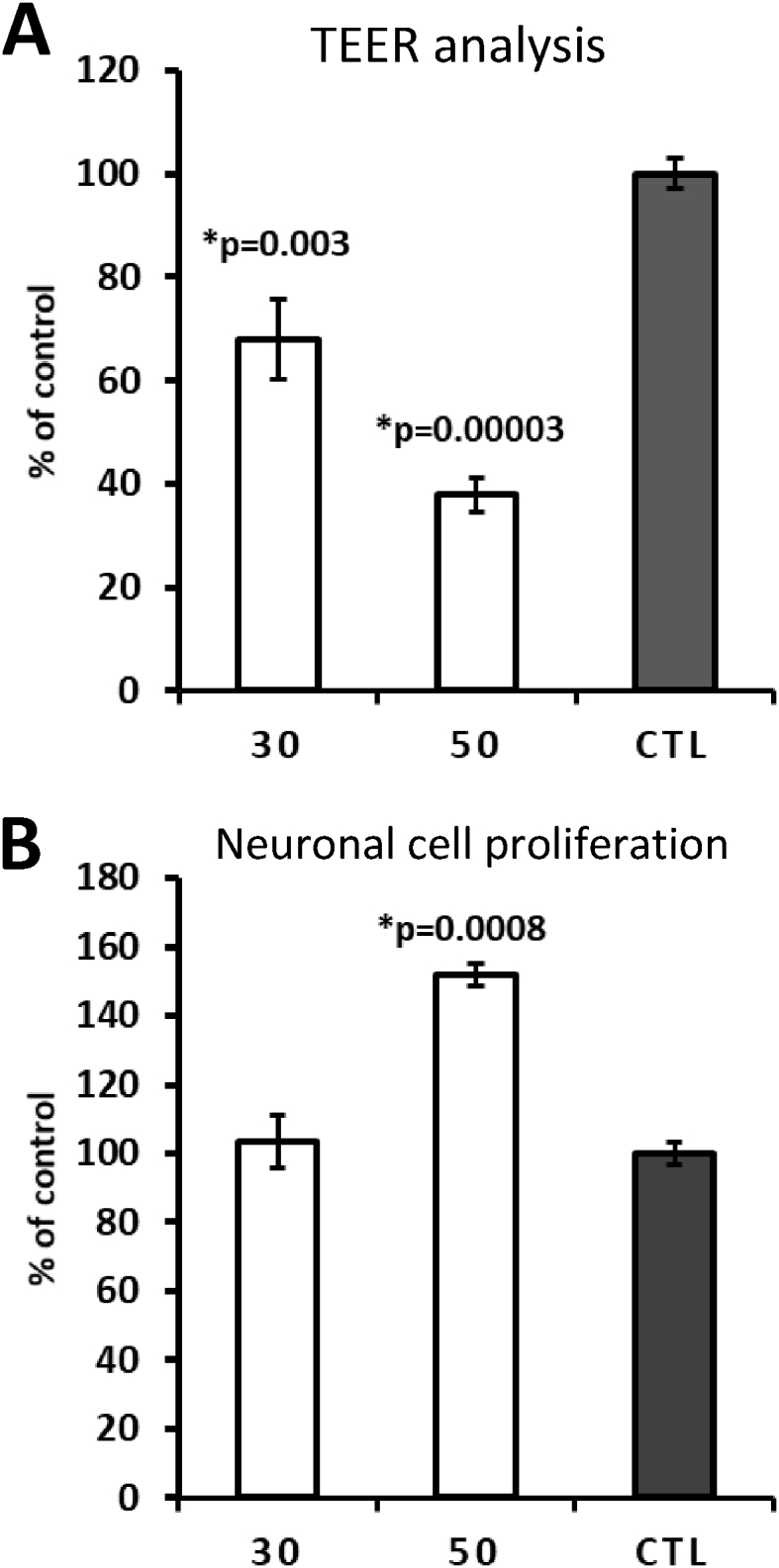
Testing higher concentrations of SMC. A) TEER measurements showing a significant concentration-dependent decrease after 30 and 50 μg/ml SMC treatment. B) On the basolateral side, a very significant increase in neuronal cell proliferation is observed after 50 μg/ml SMC treatment. The data are mean ± SE (n = 4). Difference between the control and treatment is significant (Student's t-test, p < 0.05).

The effects of collagen peptides have already been reported *in vitro* and *in vivo* (Han et al., 2015; Koyama, 2016; Subhan et al., 2017; Xu et al., 2015). However, the current study compared the effects of a hydrolyzed collagen SMC versus trypsinized SMC on distinct dual-chamber models *in vitro*, including the triple co-culture model of the BBB (Fig. 8). In this model, SMC caused a 50% increase in neuronal SH-SY5Y cell proliferation (Fig. 8C) growing on the basolateral side. This data indicates the ability of SMC to permeate the BBB and promote neurogenesis. It is worth noting that the TEER in this model was not transepithelial; rather, Transendothelial Electrical Resistance, since we used HUVEC endothelial cells to grow on inserts. Moreover, in the same way as in the GEA model, TSMC caused an increase on TEER (i.e., an increase in cellular tightness) (Fig. 8B), which could explain why TSMC was not able to affect neuronal cell proliferation on this BBB model. SMC *per se* caused a significant decrease in TEER (Fig. 8B), understanding that the cellular monolayer permeability was increased by the treatment. To confirm that SMC and TSMC do not cause any harm to the BBB cells (HUVEC and U87MG), we performed MTT analysis also on these cells, growing them separately as a monolayer in the 96-well plate. As demonstrated in Fig. 10, TSMC caused a slight increase (7%) in astrocyte U87MG cell viability. However, on HUVEC cells, SMC caused this slight increase (10%) in cell viability. Nevertheless, the decrease in TEER values observed after treatment with SMC was not because of any toxic effect on BBB cells, indicating that the BBB was indeed more permeable after SMC treatment, which relies on its supposed ability to act directly on tight junctions rather than being cytotoxic. In the same way, a study has suggested that the paracellular pathway is responsible for the transepithelial transport of collagen peptides (Shimizu et al., 2010). It has also demonstrated—using Caco-2 monolayers—that fish collagen peptides (FCP) with low molecular size are most efficiently transported across the cell monolayers in comparison with those with medium- and maximum-sized FCP (Shimizu et al., 2010). In our assays, we observed that the size indeed facilitated SMC to reach the neuronal cells growing below on the basolateral side. Beyond that and besides the observed decrease on TEER, 15 μg/ml SMC was not able to cause any significant effect on the neuronal cell proliferation in the basolateral side on the GBA model. The lack of effect could be explained by the difference in the cellular tightness between Caco-2 and HUVEC cells. Thus, we may infer that in the case of the GBA model, a higher concentration of SMC is necessary to obtain a significant effect on neuronal cell proliferation growing in the basolateral side. To assess this hypothesis, additional concentrations of 30 and 50 μg/ml SMC were tested in another assay (Fig. 11A and B). It was observed that 50 μg/ml is indeed the concentration needed to cross the gut-barrier and induce neurogenesis. Importantly, assuming that the concentration-response curve showed 30 μg/ml SMC to be neurotoxic, it is supposed that not all SMC derived molecules are able to pass through the barrier. On the other hand, in the GEA model, the given concentration of 15 μg/ml was sufficient to pass the gut-barrier of Caco-2 and induce the increase in BJ cell proliferation in the basolateral side.

## 4 Conclusions

In this study, we managed to demonstrate important *in vitro* mechanisms of action caused by the hydrolyzed collagen SMC, which has already proved its clinical effects in the clinic:

• SMC was able to induce adhesion specifically in cells that do not possess good attachment ability.
• SMC was able to induce migration, accelerating wound closure in cells from the normal human epithelium and endothelium.
• For the first time, the effect of SMC was demonstrated in comparison with its trypsinized version (TSMC) on the permeability of gut-like and blood-brain-like barriers, where SMC caused a significant increase on their permeability. SMC was shown to increase the proliferation of neuronal (neurogenesis) and epithelial cells growing on the basolateral side of the BBB and the GEA models, respectively. Concerning the GBA model, we concluded that the main barrier to be crossed by SMC is not the BBB but the gut, because of its cellular junction tightness. The small molecular weight of SMC apparently allows lateral diffusion in the lipid bilayer, which is fluid (Watson, 2015).

Importantly, this is the first time that elementary assays like these are performed to demonstrate the function of a collagen product *in vitro*. We also conclude that these models can provide a feasible and translational significant *in vitro* screening tool for collagen and probably other functional foods and nutritional supplements.

## Declarations

### Author contribution statement

Marcel Benadiba: Conceived and designed the experiments; Performed the experiments; Analyzed and interpreted the data; Contributed reagents, materials, analysis tools or data; Wrote the paper.

Raphael Serruya: Performed the experiments; Contributed reagents, materials, analysis tools or data.

Yehoshua Maor: Conceived and designed the experiments; Analyzed and interpreted the data; Contributed reagents, materials, analysis tools or data; Wrote the paper.

### Funding statement

This work was supported by the Generation Joy LLC. Further information is available at http://www.generationjoy.com.

### Competing interest statement

The authors declare no conflict of interest.

### Additional information

No additional information is available for this paper.
